# Targeting Reactive Carbonyl Species with Natural Sequestering Agents

**DOI:** 10.3390/molecules21030280

**Published:** 2016-02-27

**Authors:** Sung Won Hwang, Yoon-Mi Lee, Giancarlo Aldini, Kyung-Jin Yeum

**Affiliations:** 1 Department of Nano Science & Mechatronics Engineering, College of Science and Technology, Konkuk University, Chungju-si 27478, Korea; swhwang@kku.ac.kr; 2 Division of Food Bioscience, College of Biomedical and Health Sciences, Konkuk University, Chungju-si 27478, Korea; yoonmilee@kku.ac.kr; 3 Interdisciplinary Research Center for Health, Konkuk University, Chungju-si 27478, Korea; 4 Department of Pharmaceutical Sciences Pietro Pratesi, Università degli Studi di Milano, via Mangiagalli 25, Milan 20133, Italy; giancarlo.aldini@unimi.it

**Keywords:** reactive carbonyl species, natural products, bioefficacy, nanotechniques

## Abstract

Reactive carbonyl species generated by the oxidation of polyunsaturated fatty acids and sugars are highly reactive due to their electrophilic nature, and are able to easily react with the nucleophilic sites of proteins as well as DNA causing cellular dysfunction. Levels of reactive carbonyl species and their reaction products have been reported to be elevated in various chronic diseases, including metabolic disorders and neurodegenerative diseases. In an effort to identify sequestering agents for reactive carbonyl species, various analytical techniques such as spectrophotometry, high performance liquid chromatography, western blot, and mass spectrometry have been utilized. In particular, recent advances using a novel high resolution mass spectrometry approach allows screening of complex mixtures such as natural products for their sequestering ability of reactive carbonyl species. To overcome the limited bioavailability and bioefficacy of natural products, new techniques using nanoparticles and nanocarriers may offer a new attractive strategy for increased *in vivo* utilization and targeted delivery of bioactives.

## 1. Introduction

Reactive oxygen species (ROS) are continuously generated through normal cell metabolism in the body [1], and are necessary for biological homeostasis [2]. However, an imbalance between oxidant production and antioxidant defense can lead to an accumulation of excess ROS which damage vulnerable targets such as unsaturated fatty acids in membranes, thiol groups in proteins and nucleic acids in DNA [3]. Thus, oxidative stress can be associated with the development and progression of various chronic diseases. In particular, elevated cytotoxic reactive carbonyl species, which are produced by the oxidation of polyunsaturated fatty acids and sugars [4], plays a crucial role in the progression of metabolic disorders such as diabetes [5] and cardiovascular diseases [6] and neurodegenerative diseases [7]. Carbonyl species are highly reactive due to their electrophilic nature, and easily react with the nucleophilic amino acids such as Lys, His and Cys, leading to the formation of protein adducts [8,9]. The formation of these protein adducts has been reported to cause irreversible cellular dysfunction [10,11].

The use of natural products that can effectively sequester reactive carbonyl species [12,13] may offer a novel strategy blocking the pathological conditions and progression of various chronic diseases. In addition, new techniques such as nanoparticles and nanocarriers that can increase the bioavailability and bioefficacy of natural products *in vivo* may open up a field for preventing oxidative stress associated chronic diseases using natural products.

However, substantial knowledge gaps still exist including: (1) what effective sequestering agents are and their mechanisms *in vivo*; (2) reactive carbonyl species etiology leading to cellular dysfunction *in vivo*; and (3) whether genetic variation affects the biological efficacy of different sequestering agents for reactive carbonyl species.

## 2. Oxidative Stress and Reactive Carbonyl Species

**Figure 1:**
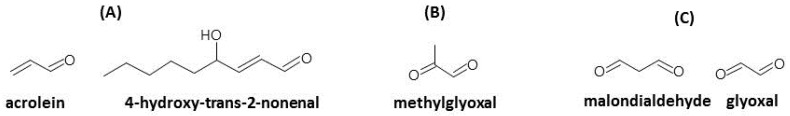
Structures of reactive carbonyl species: α,β-unsaturated aldehydes (**A**); keto-aldehyde (**B**) and di-aldehydes (**C**).

Reactive carbonyl species can be classified into three groups: (1) α,β-unsaturated aldehydes such as 4-hydroxy-*trans*-2-nonenal (HNE) and acrolein; (2) keto-aldehydes such as methylglyoxal and (3) dialdehydes such as glyoxal and malondialdehyde as shown in Figure 1 [14].

Proteins represent the most studied target of reactive carbonyl species and the corresponding reaction products are named advanced glycation end products (AGEs) when the attacking RCS is derived from sugar, and called advanced lipoxidation end products (ALEs) when it derives from lipids. AGEs and ALEs share similar structural and biological properties. For example, both consist of non-enzymatic, covalently modified proteins and oxidative stress is often (but not always) involved in the mechanism of their formation. Moreover some AGEs and ALEs have the same structure, since they arise from common precursors, as in the case of carboxymethyllysine (CML) which is generated by glyoxal that is formed by both lipid and sugar oxidative degradation pathways [14].

HNE represents one of the most abundant and toxic reactive carbonyl species, which is generated via β-cleavage of hydroperoxide derived from ω-6 polyunsaturated fatty acids such as linoleic acid and arachidonic acid [4]. HNE can give covalent adducts with the protein nucleophilic side chains, namely, the cysteine thiol group, the lysine ε-amino group, and the histidine imidazole ring [15]. AGEs are generated by the covalent reaction of reactive carbonyl species derived from sugar oxidation such as glyoxal, methylglyoxal and 3-deoxyglucosone with the nucleophilic protein sites, as well as by the condensation of the carbonyl group of reducing sugars with the primary amino group of the lysine side chain or of the protein N-terminus [16]. Covalent modifications of AGEs and ALEs can induce a functional disorganization of proteins since covalent modification causes the protein to undergo a conformational change, undergo catalytic site distortion or impairment of the function of the protein itself. AGEs and ALEs can further modify proteins by inducing signal transduction leading to cellular damage [16]. The interaction of AGEs and possibly ALEs with receptors for advanced glycation end products (RAGE) leads to NFκB activation which is known to cause the production of inflammatory cytokines including IL-1, IL-6 and TNF-α. RAGE has even been proposed as a master switch to turn on the proinflammatory response into a cellular dysfunction [17]. RAGE activation also induces the production of excessive mitochondrial ROS thereby leading to mitochondrial superoxide accumulation [18]. In that sense, it is reasonable to consider that blocking ROS production is an appropriate strategy in order to reduce mitochondrial superoxide accumulation in diabetic patients [18]. It is interesting to note that an elevation of keto-aldehydes such as methylglyoxal in type II diabetic patients, and its reduction by the diabetic drug, metformin, has been observed [19]. In addition, methylglyoxal has been reported to play a critical role in diabetic complication, nephropathy [20].

The association of AGEs/ALEs with chronic diseases and the mechanism of interaction between AGEs and RAGE have been identified partially. However, effective AGEs/ALEs sequestering agent which can block the AGEs-RAGE interaction, and its ability to inhibit inflammatory responses and related pathologic progression of chronic diseases *in vivo*, is not yet known.

## 3. Implication of Reactive Carbonyl Species on Metabolic Disorders

Evidence is mounting that oxidative stress and protein carbonylation damage induced by reactive carbonyl species are involved in metabolic disorders such as dyslipidemia, insulin resistance, vascular and renal diseases [10,21,22,23]. Table 1 presents targets of different reactive carbonyl species to prevent metabolic disorders in cells, animal models and humans. Various cell lines such as muscle cells [24], pancreatic β-cells [25] and human mesangial cells [26] have been studied to determine various drug actions to block reactive carbonyl species, AGEs, RAGE and protein carbonyls, thereby preventing metabolic disorders. In addition, various animal models utilized to determine the effect of blocking reactive carbonyl species, AGEs and RAGEs on metabolic disorders and their complications. Zucker rats [27] and ApoE null mice [28] have been employed to evaluate the reactive carbonyl species sequestering actions of carnosine and its derivatives, respectively. In addition, streptozotocin induced diabetic rats [29], CCl_4_-injected [30] or high fructose-fed [31] Wistar rats, and methylglyoxal injected Dahl salt-sensitive rats [32] were used to target reactive carbonyl species, AGEs, RAGE and/or protein carbonyls in diabetic complications. Furthermore, in humans, type II diabetic patients were studied for their elevated methyl glyoxal levels [19] as well as RAGE expression [33]. Nonetheless, reactive carbonyl species and their adducts are closely associated with the progression of metabolic disorders and such complications and can be alleviated by reactive carbonyl species sequestering agents.

## 4. Implication of Reactive Carbonyl Species on Neurodegenerative Diseases

The carbonylation of histidine and lysine residues of apolipoprotein B (apoB-100) in low-density lipoproteins (LDL) has been reported to be implicated in the formation of foam cells [34]. Interestingly, modified LDL by HNE has also been found to cause a significant elevation of β-amyloid fibrillogenesis [7], suggesting an involvement of reactive carbonyl species in neurodegenerative diseases such as Alzheimer’s disease. Table 2 lists several *in vitro* and *in vivo* studies targeting reactive carbonyl species for preventing neurodegenerative diseases.

Reactive carbonyls species and protein carbonyls have been reported to induce neuronal damage, and bioactives such as epigallocatechin gallate [35] as well as notoginsenoside [36] alleviated such damage in neuronal cell lines. In addition, various studies utilizing animal models also presented consistent results. Animal studies utilizing d-galactose-injected C57BL/6 mice [37], ^56^F-irradiated C57BL mice [38], high cholesterol-fed C58BL/6 mice [39], SAMP8 mice [40], streptozocin-injected Wistar rats [41], 2,2′-Azobis(2-amidinopropane) dihydrochloride (AAPH) or Fe^2+^/H_2_O_2_-injected Monglian gerbils [42] indicated involvement of reactive carbonyl species and protein carbonyls on neuronal damage and Alzheimer’s disease. These studies indicated that such diseases were ameliorated by blocking oxidative damaged caused by reactive carbonyl species. In cancer patients, mercaptoethane sulfonate has been reported to be used for reducing oxidative stress induced by doxorubin treatment [43].

**Figure 2:**
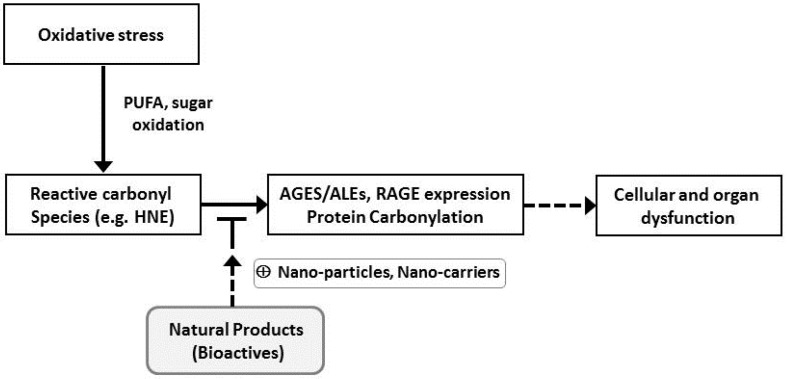
Hypothetical cytotoxic reactive carbonyl species sequestering action of natural products. PUFA, polyunsaturated fatty acid; HNE, 4-hydroxy-*trans*-2-nonenal; AGEs/ALEs, advanced glycation end product/advanced lipoxidation end products; RAGE, receptor for advanced glycation end products.

Unfortunately, an effective preventive strategy for chronic diseases such as metabolic disorders and neuronal diseases is currently lacking. However, identification of natural products that are able to directly or indirectly detoxify the reactive carbonyl species may offer new therapeutic agents to combat such diseases. The hypothetical sequestering mechanism of natural products for cytotoxic reactive carbonyl species is presented in Figure 2.

## 5. Analytical Techniques for Identifying Reactive Carbonyl Species Sequestering Agents

In order to determine the sequestering actions of natural products on reactive carbonyl species, a reliable and accurate method that can identify these actions of bioactives in complex mixture is required. Several approaches have been reported for identifying such compounds as shown in Table 3.

A spectrophotometric assay has been widely used to analyze chromophore containing reactive carbonyl species such as α,β-unsaturated aldehydes directly or through a derivatization process for unconjugated reactive carbonyl species such as malondialdehyde, glyoxal, and methylglyoxal [44]. The reactive carbonyl species quenching activity can be determined by the disappearance of aldehyde in the presence of a compound of interest. An integration of HPLC with UV analysis was also utilized to increase specificity [45]. However, these types of approaches cannot be applied to mixed compounds containing natural products. In addition, by-products can be produced in the process of sample preparation resulting in the loss of accuracy.

Determination of the formation of AGEs/ALEs by incubating reactive carbonyl species with a target protein in the presence of a potential quencher has also been used to identify sequestering agents against reactive carbonyl species. The formation of AGEs/ALEs can be determined by increased molecular weight using NMR spectroscopy [46] or Western blot [47]. However, these types of assay can be time consuming, expensive and cannot be quantitative.

A new approach using high resolution mass spectrometry was reported to test the ability of natural compounds inhibiting protein carbonylation induced by reactive carbonyl species [48]. It consists of incubating ubiquitin with 4-hydroxy-*trans*-2-nonenal (HNE), in the presence and absence of natural products. After incubation, the reaction can be stopped and analyzed for reaction metabolites using high-resolution mass spectrometry. This approach has been validated by measuring the effect of well-known reactive carbonyl species sequestering agents, such as aminoguanidine, pyridoxamine, hydralazine and carnosine. A highly reproducible mass spectrometric method was also found suitable for testing reactive carbonyl species sequestering ability of complex mixtures such as plant extracts, thus furnishing a methodological approach for identifying novel natural compounds that are effective as reactive carbonyl species sequestering agents. It should be noted that an approach permiting evaluation of overall quenching activity of complex mixtures open limits identification of responsible component(s) for the quenching activity. Characterization of sequestering agent in natural products require further analysis coupled with informatics approach.

## 6. Reactive Carbonyl Species Sequestering Actions of Natural Products

Convincing evidence is accumulating that a higher consumption of fruits and vegetable reduces all-cause mortality and cardiovascular mortality [49], whereas no beneficial [50,51] and even harmful effects [52,53] of multivitamins or antioxidant supplements against chronic diseases has been observed. Considering several natural products have been reported for their reactive carbonyl species sequestering action, natural products that can effectively sequester reactive carbonyl species can be a potential preventive strategy against such chronic diseases.

### 6.1. Histidine-containing Dipeptides

*In vitro* studies have shown that histidine dipeptides such as carnosine (β-alanyl-l-histidine) and anserine (β-alanyl-l-methylhistidine) effectively detoxifies HNE by forming unreactive adducts [54]. Notably, histidine, which is one of the most reactive nucleophilic residues in protein, is a primary reaction site of HNE adduction [55]. Histidine-dipeptides such as carnosine supplementation has been reported to significantly reduce the development of dyslipidemia, hypertension and renal injury by reducing the extent of protein carbonylation and glycation in Zucker obese rats [27]. In addition, histidine-dipeptides have proven to be beneficial in various animal models characterized for systemic oxidative and/or glycative stress [27,56,57,58,59,60]. There is also compelling evidence that histidine-dipeptides mediate their health-promoting effects by decreasing the levels of AGEs/ALEs thereby blocking damage of AGEs/ALEs-RAGE in these animal models.

Gene-nutrient interactions may result in different bioefficacy of supplements according to the genetic background of individuals. Such interactions have been reported in vitamin C-glutathione S-transferase [61] and vitamin E-haptoglobin [62]. The association of low serum carnosin concentration with diabetic nephropathy has also been reported. It was found that carnosinase encoding gene, CNDP1, linked with the late onset of complications for people with diabetes [56,63]. More specifically individuals who have the 5-6, 5-7, 6-6, and 6-7 alleles of the CNDP1 gene had elevated serum carnosinase activity. Diabetic patients with the 5-5 allele, which accounted for about 1/3 population in this study, were found to be less susceptible to renal complication [56,63]. It is reasonable that the higher expression of carnosinase increases carnosine degradation leading to a lesser degree of renal protection by carnosine. Although such a hypothesis should be verified further in human studies, nutrient-gene interaction is an area needs to be explored for the understanding of bioefficacy of natural products.

### 6.2. Plant Products

More recently, black rice with giant embryos rich in GABA, anthocyanin, γ-oryzanols, α-toco-pherol and α-tocotrienols has been reported to suppress hyperlipidemic and hyperinsulinemic responses in *ob/ob* mice [64]. Although one should be cautious when extrapolating results from animal studies to humans, identification of such activity in natural products can provide more targeted preventive strategy against chronic diseases. In addition, the effects of green coffee bean extract and procyanidins from *Vitis vinifera* on protein carbonylation have been demonstrated using the newly developed mass spectrometry approach [48]. These two extracts are reported to have an effective inhibition of HNE induced ubiquitin carbonylation in a dose-dependent manner *in vitro*.

Screening of natural products for reactive carbonyl species quencher is the first step to identify potential candidates for a targeted strategy preventing oxidative stress associated chronic diseases. However, several further steps need to be made including understanding of gene-nutrient interactions, and increasing the limited bioavailability and bioefficacy of natural products.

## 7. Nanotechnologies for Bioavailability and Bioefficacy of Natural Products

**Figure 3:**
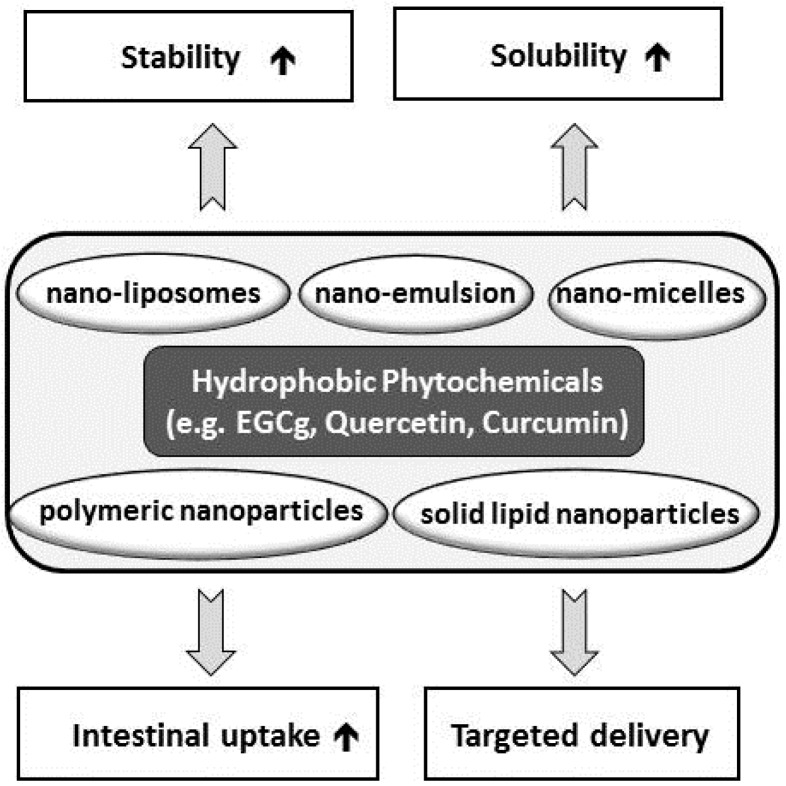
Application of nano-technologies improving bioavailability of natural products. EGCg, epigallocatechin gallate.

The major hindrance of oral intake of natural products, including phytochemicals, is their limited bioavailability due to their poor solubility, instability, and negligible intestinal absorption. Considering mega-doses is not a solution to address the limited bioavailability of such natural products, so development of effective delivery systems improving bioavailability and bioefficacy is a key issue for nutraceutical research. In fact, applications of nanotechnology to improve bioavailability and bioactivity of diet-derived phytochemicals have been reported recently [65,66,67]. Biocompatible and biodegradable nanoparticles such as nanoemulsions, nanoliposomes, and nano-carriers are reported to resolve the limited bioavailability of phytochemicals, as summarized in Figure 3 [65].

It has been reported that nanoliposomes enhanced the stability of epigallocatechin gallate (EGCG) [68], and increased its antioxidant activity [69]. Nanomicelles were applied to overcome the low bioavailability of quercetin, which is a plant-derived hydrophobic flavonol [70,71]. In addition encapsulation of quercetin is reported to maintain free quercetin levels in blood and target tissues by delaying its metabolism [72]. Oral bioavailability [73] and its bioefficacy [74] of hydrophobic curcumin has also been reported to be dramatically improved by application of nanotechnology.

**Figure 4:**
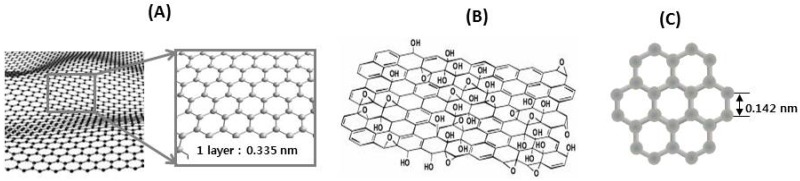
Structures of graphene (**A**); graphene oxide (**B**) and grapheme quantum dot (**C**).

In addition, advances in technology for nanomaterials [75,76] may also provide great potentials for improving bioavailability and bioefficacy of natural products. The recent discovery of graphene has spurred on various research approaches for targeted delivery of active compounds. Graphene is a single atom thick layer of sp^2^-hybridized carbon atoms arranged in a honeycomb two dimensional (2D) crystal lattice [77]. Owing to its unique atomic structure, graphene has flexible physical and chemical properties, large surface area and biocompatibility, fast mobility and outstanding electrical conductivity [78]. These properties make graphene an ideal material for a variety of applications including quantum mechanics and engineering of biomaterials such as new generation biosensors [79], probes for biological imaging [80] and nanocarriers for drug delivery [81]. Among various nano-materials explored for the last two decades for drug delivery, graphene, graphene oxide (GO) and grapheme quantum dots (Figure 4) have emerged as new competitive nanocarriers for drug delivery and possibly natural products delivery.

In order to achieve the successful design of nanocarriers for natural products, various issues such as optimizing loading capacity, improving biocompatibility, eliminating toxicity, and controlling release needed to be resolved.

## 8. Summary

Even though the marked increase of life expectancy in recent years can be considered one of our society’s greatest achievements, unhealthy eating and lifestyle habits can cause a concomitant dramatic rise of chronic and neurodegenerative diseases. In an effort to reduce the prevalence of oxidative stress associated such chronic diseases, various strategies including consuming multivitamins and antioxidant supplementation have been utilized. Unfortunately, supplementation with high doses of single compounds such as vitamin E failed to show any beneficial effect against chronic diseases and even had harmful effects such as an increased risk of mortality. Unlike well-known antioxidants such as vitamin E, bioactives in natural products that can effectively sequester cytotoxic reactive carbonyl species can provide more targeted action against oxidative stress associated pathologic conditions. Thanks to the recent development of new techniques utilizing high resolution mass spectrometry, reactive carbonyl species sequestering actions of natural products have begun to be identified. In addition, nanotechnologies including nanoparticles and nanocarriers are being explored in order to overcome the limitation of bioavailability and bioefficacy of natural products in humans.

## Figures and Tables

**Table 1:** Studies targeting reactive carbonyl species to prevent metabolic disorders.

Metabolic Disorders	Targeting RCS	Tested Agent	Model	Ref.
**Cell Studies**				
Insulin resistance	HNE, Protein carbonyls	D3T, NAC, AGD, SAM	Gastrocnemius muscle, muscle cells (L-6)	[24]
Insulin resistance	AGEs, Protein carbonyls	AGD, Pyridoxamine	Pancreatic β-cells (HIT-T15)	[25]
Diabetic nephropathy	RAGE	Glucagon-like peptide 1	Human mesangial cells	[26]
**Animal Studies**				
Dyslipidemia	HNE, AGEs	Carnosine	Zucker Fa/Fa rats	[27]
Atherosclerosis Renal disease	HNE, ALEs	D-carnosine octylester	ApoE null mice (HFD)	[28]
Diabetic atherosclerosis	RCS, AGEs, ALEs, RAGE	LR-90	Streptozotocin induced diabetic rats	[29]
Liver damage	AGEs, RAGE, protein carbonyls	Glycyrrhizi	High fructose-fed Wistar rats	[31]
Liver/renal toxicity	RAGE, Protein carbonyls	Peach	CCl_4_ injected Wistar rats	[30]
Diabetic nephropathy	RAGE	Candesartan	MG injected Dahl salt-sensitive rats	[32]
**Human Studies**				
Diabetes and complication	RAGE	Simvastatin	Type 2 diabetic patients	[33]
Diabetes	MG	Metformin	Type 2 diabetic patients	[19]

RCS, reactive carbonyl species; HNE, 4-hydroxy-trans-2-nonenal; D3T, 3*H*-1,2-dithiole-3-thione; NAC, *N*-acetyl-cysteine; AGD, aminoguanidine; SAM, *S*-adenosylmethionine; AGEs: advanced glycation end products; RAGE, receptor for advanced glycation end products; ALEs, advanced lipoxidation end products; HFD, high fat diet; MG, methyl glyoxal.

**Table 2:** Studies targeting reactive carbonyl species to prevent neurodegenerative diseases.

Neurodegenerative Diseases	Targeting RCS	Tested Agent	Model	Ref.
**Cell Studies**				
Neuronal damage	MDA, AGE-RAGE, Protein carbonyls	EGCG	AGE treated SH-SY5Y cells	[35]
Neuronal damage	MDA, Protein carbonyls	Notoginsenoside	H_2_O_2_ treated PC12 cells	[36]
**Animal Studies**				
Brain inflammation	AGEs, RAGE, Protein carbonyls	Ursolic acid	D-galactose injected Kunming mice	[37]
Neuronal damage	MDA, Protein carbonyls	Melatonin	^56^F-irradiated C57BL mice	[38]
Alzheimer’s disease	AGEs, Protein carbonyls	Troxerutin	High cholesterol fed C57BL/6 mice	[39]
Alzheimer’s disease	HNE	Antisense oligonucleotide	SAMP8 mice	[40]
Alzheimer’s disease	HNE, Protein carbonyls	Curcumin	Streptozotocin-injected Wistar rats	[41]
Alzheimer’s disease	HNE, Protein carbonyls	Ferulic acid ethyl ester	AAPH or Fe^2+^/H_2_O_2_ injected Mongolian gerbils	[42]
**Human Studies**				
Cognitive dysfunction	HNE, Protein carbonyls	2-Mercaptoethane sulfonate	doxorubicin-received patients	[43]

RCS, reactive carbonyl species; AGEs: advanced glycation end products; RAGE, receptor for advanced glycation end products; EGCG, epigallocatechin gallate; AAPH, 2,2′-Azobis(2-amidinopropane) dihydrochloride; HNE, 4-hydroxy-trans-2-nonenal.

**Table 3:** Analytical techniques identifying reactive carbonyl species sequestering agents.

Analytical Techniques	Advantages	Disadvantages	Ref
Spectrophotometry	Simple Fast	No specificity No application for complex mixture	[44]
HPLC	Limited specificity	No application for complex mixture Produce by-product	[45]
NMR spectroscopy	Molecule identification	No qunatitation Expensive Require large quantity of sample	[46]
Western blot	Semiqunatitative	Time consuming	[47]
LC-MS	Quantitative Complex mixture analysis	Molecule identification require further analysis	[48]

HPLC, high performance liquid chromatography; NMR spectroscopy, nuclear magnetic resonance spectroscopy; LC-MS, high resolution mass spectrometry.
